# miR-107 regulates tumor progression by targeting NF1 in gastric cancer

**DOI:** 10.1038/srep36531

**Published:** 2016-11-09

**Authors:** Shizhi Wang, Gaoxiang Ma, Haixia Zhu, Chunye Lv, Haiyan Chu, Na Tong, Dongmei Wu, Fulin Qiang, Weida Gong, Qinghong Zhao, Guoquan Tao, Jianwei Zhou, Zhengdong Zhang, Meilin Wang

**Affiliations:** 1 Department of Environmental Genomics, Jiangsu Key Laboratory of Cancer Biomarkers, Prevention and Treatment, Collaborative Innovation Center For Cancer Personalized Medicine, Nanjing Medical University, Nanjing, China; 2 Department of Genetic Toxicology, the Key Laboratory of Modern Toxicology of Ministry of Education, School of Public Health, Nanjing Medical University, Nanjing, China; 3 Key Laboratory of Environmental Medicine Engineering, Ministry of Education, School of Public Health, Southeast University, Nanjing, China; 4 Core Laboratory, Nantong Tumor Hospital, Nantong, China; 5 Department of General Surgery, The Affiliated Jiangning Hospital of Nanjing Medical University, Nanjing, China; 6 Department of General Surgery, Yixing Cancer Hospital, Yixing, China; 7 Department of General Surgery, The Second Affiliated Hospital of Nanjing Medical University, Nanjing, China; 8 Department of General Surgery, Huai-An First People’s Hospital Affiliated to Nanjing Medical University, Huai-An, China; 9 Department of Molecular Cell Biology and Toxicology, Cancer Center, School of Public Health, Nanjing Medical University, Nanjing, China

## Abstract

Our previous genome-wide miRNA microarray study revealed that miR-107 was upregulated in gastric cancer (GC). In this study we aimed to explore its biological role in the pathogenesis of GC. Integrating *in silico* prediction algorithms with western blotting assays revealed that miR-107 inhibition enhanced NF1 (neurofibromin 1) mRNA and protein levels, suggesting that NF1 is one of miR-107 targets in GC. Luciferase reporter assay revealed that miR-107 suppressed NF1 expression by binding to the first potential binding site within the 3′-UTR of *NF1* mRNA. mRNA stable assay indicated this binding could result in *NF1* mRNA instability, which might contribute to its abnormal protein expression. Functional analyses such as cell growth, transwell migration and invasion assays were used to investigate the role of interaction between miR-107 and its target on GC development and progression. Moreover, We investigated the association between the clinical phenotype and the status of miR-107 expression in 55 GC tissues, and found the high expression contributed to the tumor size and depth of invasion. The results exhibited that down regulation of miR-107 opposed cell growth, migration, and invasion, whereas NF1 repression promoted these phenotypes. Our findings provide a mechanism by which miR-107 regulates NF1 in GC, as well as highlight the importance of interaction between miR-107 and NF1 in GC development and progression.

Gastric cancer (GC) is the fourth most common cancer worldwide, with a total of 989,600 new cases and 738,000 deaths occurred in 2008^1^. It is well accepted that gastric carcinogenesis is a multistep and multifactorial process involving genetic and epigenetic alterations in oncogenes and tumor suppressor genes^2^,^3^.

miRNAs, an important component of epigenetic mechanisms, comprise species of short noncoding RNA and post-transcriptionally modulate gene expression by negatively regulating the stability or translational efficiency of their target mRNAs^4^. miRNAs play a crucial role in a broad range of physiological and pathological process, and their aberrant expression has been observed in a variety of diseases, including neurodegenerative disease^5^, diabetes^6^ and tumors^7^. Increasing studies suggest miRNAs are promising biomarkers for cancer diagnosis, prognosis and therapeutic targets^7^,^8^.

Our previous genome-wide miRNA microarray study has identified 12 miRNAs including miR-107 significantly dysregulated in GC^9^. miR-107 has been reported to play a vital role in cell division, metabolism, stress response, and angiogenesis. Dysregulated of miR-107 is involved in multiple tumors development and progression^10^. miR-107 functions as a tumor suppressor by inducing cell cycle arrest in lung cancer and glioma via down-regulating the expression of CDK6^11^,^12^. However, miR-107 serves as oncogene for promoting tumor invasion and metastasis in breast and gastric cancer through targeting DICER1^13^,^14^. The role of miR-107 in tumors is contradictory and needed to be further addressed. In this study, we sought to determine the role of miR-107 underlying the pathogenesis of GC.

## Methods and Materials

### Cell lines and reagents

Human GC cell lines SGC7901 and MGC803 were purchased from the Shanghai Institute of Biochemistry and Cell Biology, Chinese Academy of Sciences (Shanghai, China). Cells were cultured in DMEM/10% fetal bovine serum and grown at 37 °C and 5% CO_2_.

### Small interfering RNA

miR-107 inhibitor and negative control (designated si-107 and NC, respectively) were chemically synthesized by GenePharma Tech (Shanghai, China). The sequences of si-107 and NC were 5′-UGAUAGCCCUGUACAAUGCUGCU-3′ and 5′-CAGUAC UUUUGUGUAGUACA-3′, respectively; all the bases were 2′-OMe modified. Small interfering RNA (siRNA) specific for NF1 (sc-36036) and non-specific control siRNA (sc-37007) were purchased from Santa Cruz Biotechnology (Santa Cruz, CA, USA). In all functional analysis, NC and si-107 were used at a concentration of 50 nmol/L, while si-NF1 and its control at 10 nmol/L.

### Plasmids construction, transient transfection and luciferase assay

**Figure 1:**
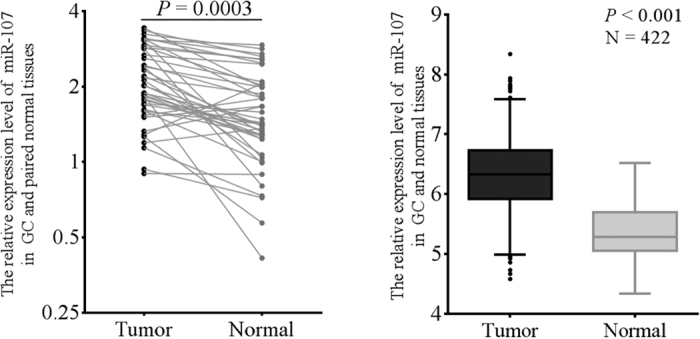
The difference of miR-107 expression between the gastric carcinoma and paired corresponding non-cancerous tissues. (**A**) The expression of miR-107 in 55 paired of gastric carcinoma and corresponding normal tissues. (**B**) The expression of miR-107 in TCGA (n = 422).

The NF1 Wild-type reporter plasmid was constructed by cloning a 490-bp DNA fragment of *NF1* 3′-untranslated region (UTR), which is containing the two predicted miR-107 target sites, into the *Hpa*I site of the pGL3-promoter vector (Promega, WI, USA; Fig. 1C, upper panel). Based on the Wild plasmid, the Mut-1, Mut-2 and Mut-both mutant constructs were made by removing the first, second and both predicted miR-107 target sites of the *NF1* 3′-UTR fragment, respectively.

The plasmids or in combination with siRNAs were transiently transfected into cells using Lipofectamine 2000 (Invitrogen, CA, USA) following the manufacturer’s protocol. As an internal standard, all plasmids were cotransfected with 10 ng pRL-SV40, which contained the Renilla luciferase gene. The pGL3-promoter vector without an insert was used as a negative control. After transfection at 48 h, luciferase activity in lysates was measured with a Dual-Luciferase Reporter Assay System (Promega, WI, USA). The reporter assay was performed with three biological replicates and three technical replicates.

### Real-time PCR analysis

Total RNA was isolated from cell lysates according to the instructions provided by the manufacturer of TRIZOL (Invitrogen, CA, USA). Reverse transcription was performed using the TaqMan MicroRNA Reverse Transcription Kit (ABI, CA, USA). The expression level of miR-107 was assessed using the specific TaqMan MicroRNA Assay kit (ABI, CA, USA), and normalized to U6. To determine the expression levels of *NF1* mRNA, the cDNA was amplified by real-time PCR with SYBR Green RT-PCR kit (Takara, Japan). The expression of *GAPDH* was used as an internal control. The following primers were used for amplification: 5′- CGAATGGCACCGAGTCTT AC-3′ (F) and 5′-GACCAGTTGGACGAGCCC -3′ (R) for *NF1*; 5′- GCACCGTCA AGGCTGAGAAC -3′ (F) and 5′- TGGTGAAGACGCCAGTGGA -3′ (R) for *GAPDH*. Real-time PCR was performed in triplicate on ABI 7900HT Real-Time PCR System (ABI, CA, USA). Relative expression was calculated using the comparative Ct method. The Real-time PCR assay was performed with three biological replicates and three technical replicates.

### Western blotting

The total cell lysates were prepared with a detergent lysis buffer (50 mM Tris, pH 7.4; 150 mM NaCl; 1% NP-40; 0.5% sodium deoxycholate; 0.1% SDS; 1 mM PMSF; and 1% phosphatase inhibitor cocktail (P2850, Sigma-Aldrich, MO, USA). Western blots were performed as previously reported^15^ using antibodies of NF1 (sc-67, Santa Cruz, CA, USA); α-tubulin, p-ERK1/2, and ERK1/2 (Cat. #2125, 9101, 9102, respectively; Cell Signaling, MA, USA).

### Cell growth assay

The transfected MGC803 cells were seeded in 96-well plates. Cell culture was continued for 24 h, 36 h and 48 h and subsequently incubated with MTT reagent (5 mg/ml) at 37 °C for 4 h. MTT assay was performed as described elsewhere^16^.

### Wound healing assay

The transfected MGC803 cells (5 × 10^5^) were cultured in 6-well plates to monolayer. The cells were then starved in serum-free medium for 12 h before a wound approximately 2 mm in width was made with a cell scraper. The wound was allowed to heal for 3 d in a fresh medium containing 1% fetal bovine serum. The wounded monolayer was photographed at the indicated day using a fluorescent microscopy (IX70, Olympus, Japan) with a 10 × objective. Wound closure was measured as a percentage of original wound width.

### Transwell assay

Transwell assay was performed using 12-well Transwell chambers (Corning Costar, Cambridge, MA, USA) with a pore size of 8 μm. For Transwell migration, Cells (1 × 10^5^) were seeded in serum-free medium in the upper chamber and incubated at 37 °C for 8 h. Afterward, the cells remained in the upper chamber were carefully removed with a cotton swab, whereas the cells having traversed to reverse face of the membrane were fixed in methanol, stained with crystal violet (0.04% in water), and counted. Transwell invasion assay was done under the same conditions as the Transwell migration assays, but in Matrigel-coated transwells (BD Biosciences, MA, USA) and incubation for 24 h.

### Statistical analysis

Quantified data are presented as mean ± SEM. The difference between two independent means was assessed by *t*-test. All *P*-values are two-sided, and *P* < 0.05 was considered to statistically significant. Statistical analyses were carried out using SAS software (V.9.1.3; SAS Institute, Cary, NC, USA) and R software (V.2.15.0; The R Foundation for Statistical Computing).

## Results

### miR-107 is upregulated in GC tissues

We detected the expression of miR-107 in 55 paired of cancer and normal tissues. The expression level of miR-107 in cancer tissues was significantly increased compared with normal tissues (*P* = 0.0003, Fig. 1A). The same result was found in TCGA (Fig. 1B). As shown in Table 1, the aberrant miR-107 expression in GC tissues was associated with tumor sizes and depth of invasion.

### NF1 is a target of miR-107

**Figure 2:**
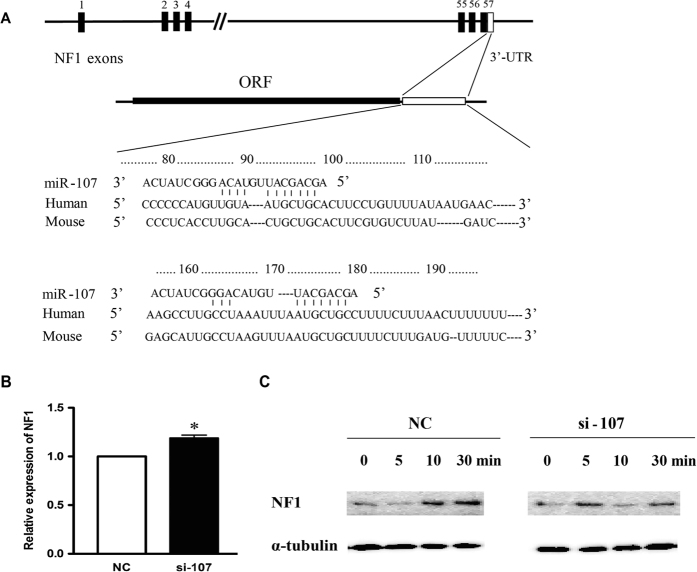
miR-107 targets NF1. (**A**) Schematic diagram of the *NF*1 3′-UTR containing two putative miR-107 binding sites. Below, sequence of mature miR-107 aligned to the two binding sites, showing evolutionary conservation in the seed-pairing sequence between human and mouse. (**B**) Down-regulation of miR-107 by si-107 resulted in an increased NF1 expression in MGC803 cells. (**C**) Down-regulation of miR-107 by si-107 resulted in an increased NF1 expression in MGC803 cells. MGC803 cells pre-treated with NC or si-107 for 48 h were serum starved for 12 h, and exposed to 10% serum for increasing lengths of time (0–30 min), and thereafter the expression of NF1 was evaluated. a-tubulin was used as internal control. *P < 0.05.

miRNA carries out its biological function via regulating the expression of its target genes through base-pairing with endogenous mRNAs. As an initial step to identify putative miR-107 targets, four commonly used algorithms (i.e., TargetScan, PicTar, Microcosm Targets v5, and miRanda) were applied to predict miR-107 target genes; and finally, there were 22 genes predicted by all four algorithms (Table 2). Among these 22 predicted target genes, *NF1* (Neurofibromin 1) stood out for the presence of two evolutionarily conserved binding sites, suggesting collaborative binding and biologically effective interaction (Fig. 2A).

NF1 is a tumor suppressor, and loss of NF1 expression has been linked to tumor development and progression^17^,^18^. To confirm that NF1 is a target of miR-107 in GC, the endogenous NF1 levels were measured in MGC803 cells at 48 h after miR-107 knockdown. The results showed that NF1 mRNA expression was significantly increased after miR-107 knockdown, compared with the negative control (Fig. 2B). Cichowski *et al.*^19^ reported that NF1 degradation could be rapidly triggered in response to growth factors and re-elevated shortly after growth factor treatment. Replicate growth factor treatment of MGC803 cells with 10% goat serum revealed that NF1 was rapidly degraded within 5 min and quickly re-elevated in the NC processing group, whereas evident degradation of NF1 was seen in 10 min in the si-107 treated group, possibly attributing to a rise in the expression of NF1 induced by miR-107 knockdown (Fig. 2C).

### miR-107 downregulates NF1 expression by directly targeting its 3′-UTR

**Figure 3:**
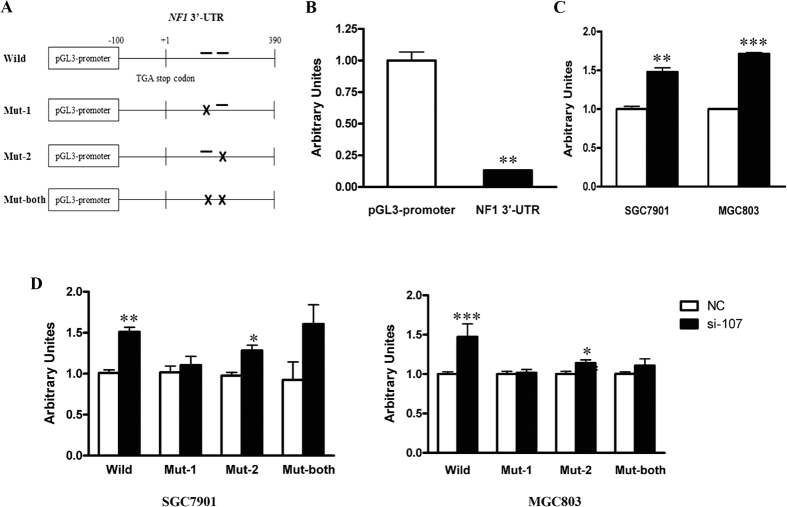
miR-107 negatively regulates NF1 expression by directly targeting its 3′-UTR. (**A**) Partial sequences of *NF1* 3′-UTR containing wild or mutated putative miR-107 target sites were fused to the firefly luciferase coding sequence. Bars, predicted miR-107 seed-pairing sequences. (**B**) The pGL3-NF1 3′-UTR reporter (Wild) and pGL3-promoter plasmids were transfected into MGC803 cells respectively to compare their luciferase activity. (**C**) The Wild plasmid was co-transfected into MGC803 cells with NC or si-107. (**D**) The Wild and three mutated plasmids (Mut-1, Mut-2 and Mut-both) were respectively co-transfected into SGC7901 and MGC803 cells with NC or si-107. Luciferase activity was normalized to a simultaneously transfected Renilla expression plasmid. *P < 0.05, **P < 0.01 and ***P < 0.001.

To establish a direct interaction between miR-107 and the 3′-UTR of *NF1*, we cloned the *NF1* 3′-UTR portion containing the two miR-107 target sites into a firefly luciferase reporter construct, designated as Wild-type Reporter (Fig. 3A), and used it for transient transfection into MGC803 cells. A significant reduction (87%) in the luciferase activity of the Wild-type Reporter was observed compared with the pGL3-promoter reporter (Fig. 3B). To evaluate whether the reduction of luciferase activity was associated with miR-107 targeting, the Wild-type Reporter was co-transfected with si-107 (miR-107 inhibitor) or NC (negative control) into the SGC7901 and MGC803 cells. The results demonstrated a significant increase in luciferase activity in both cell lines (1.48-fold for SGC7901 and 1.71-fold for MGC803) treated with si-107 compared with their NC-treated counterparts (Fig. 3C), indicating a direct interaction between miR-107 and the *NF1* 3′-UTR. As mentioned above, there are two potential miR-107 target sites within the *NF1* 3′-UTR. In order to identify the *bona fide* miR-107 target site, we mutated the first, second and both predicted target sites of the Wild-type Reporter, designated as Mut-1, Mut-2 and Mut-both reporter. Likewise, the three mutated reporters were co-transfected with si-107 or NC into MGC803 and SGC7901 cells, respectively, while the Wild-type Reporter was used as a positive control. The results revealed that in both cell lines, the luciferase activities were only increased in the cells co-transfected with the Wild and Mut-2 reporters after miR-107 knockdown compared with their NC-treated counterparts (Fig. 3D), indicating that the first predicted target site is the authentic miR-107 target site.

### Regulation by miR-107 results in *NF1* mRNA degradation

**Figure 4:**
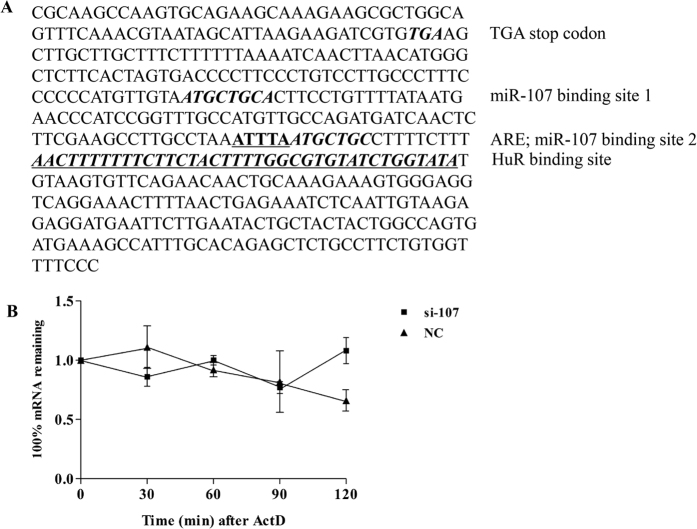
miR-107 negatively regulates NF1 expression by degrading its mRNA stability. (**A**) The predicted ARE element and HuR binding site within the *NF1* 3′-UTR. (**B**) mRNA stability of NF1 in MGC803 cells treated with NC or si-107was measured by real-time PCR.

*NF1* 3′-UTR sequence analysis revealed that there is an AU-rich element (ARE element, ATTTA) adjacent to the second predicted miR-107 target site (Fig. 4A). It was reported that miRNA could regulate the mRNA stability through binding to the ARE element in the 3′-UTR of mRNA^20^. In order to determine whether the increased expression of *NF1* mRNA was a consequence of the enhanced mRNA stability after miR-107 knockdown, we measured the mRNA stability of *NF1*. As shown in Fig. 4B, the remaining *NF1* mRNA expression was more in the si-107 treated group than in NC-treated group (1.08 ± 0.11 vs. 0.66 ± 0.09, *P* < 0.05) after ActD treatment for 2 h, implying that si-107 treatment significantly increased the stability of *NF1* mRNA in contrast with the negative control (NC) and miR-107 may have an effect on *NF1* mRNA decay and subsequent protein expression.

### miR-107 regulates cell proliferation, migration and invasion by targeting NF1

**Figure 5:**
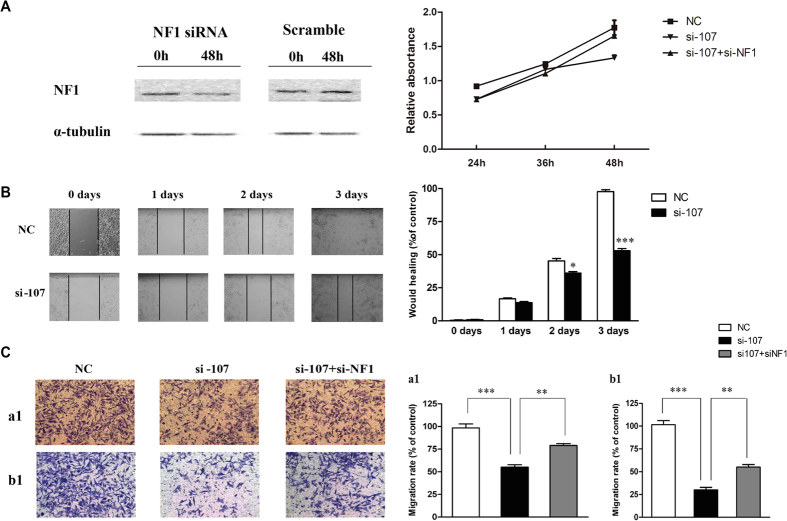
miR-107 knockdown inhibits cell growth, migration and invasion through NF1 upregulation. (**A**) MGC803 cells were transfected with NC or si-107, or si-107 in combination with si-NF1. At 48 h post-transfection, cells were seeded in 96-well plates and their growth was evaluated by MTT assay at indicated time points (right panel). Left panel, the interference effect of si-NF1 on NF1 expression. (**B**) MGC803 cells pre-treated with si-107 or NC for 48 h migrated into the wounded area. Left panel shows representative images; right panel shows the quantification of migration rate. (**C**) Transwell migration (a) and invasion (b) assays were performed in MGC803 cells pre-treated with NC, si-107, or si-107 in combination with si-NF1 for 48 h. Means ± SEM from three independent experiments. *P < 0.05, **P < 0.01, and ***P < 0.001.

It has reported that NF1 acts as a tumor suppressor to arrest cell growth, migration, and invasion^18^,^21^. We next examined the role of the interaction between miR-107 and NF1 on cell growth, migration, and invasion of GC cells. Apart from si-107 and its negative control, the MGC803 cells were further treated with si-NF1 (Fig. 5A, left panel) to evaluate whether the biological effect of miR-107 is through directly targeting NF1. The cell growth assay indicated that the cells treated with si-107 illustrated a moderate reduction in cell growth (25%) compared with its negative control (Fig. 5A, right panel). Furthermore, the cells with miR-107 knockdown displayed a significant reduction in cell migration using wound healing (46%, Fig. 5B) and Transwell migration (44%; Fig. 5C, panel a) assays. A strong decrease in invasive ability of the cells transfected with si-107 was also observed by Transwell invasion assay (70%; Fig. 5C, panel b). It was noteworthy that si-NF1 could reverse inhibition of cell proliferation (Fig. 4A, right panel), migration and invasion (Fig. 5, panel a and b, respectively) by miR-107 knockdown, indicating that miR-107 exerted its function by directly targeting NF1.

## Discussion

Recent study indicates that the role of miR-107 in tumor development and progression is contradictory and differs in a context-dependent manner. miR-107 could function as tumor suppressor gene by inducing cell cycle arrest in lung cancer and glioma^11^,^12^, whereas serve as oncogene for promoting tumor invasion and metastasis in breast and gastric cancer^13^,^14^.

In GC, Li *et al.*^14^ found that miR-107 was upregulated in GC and promoted tumor invasion and metastasis by negatively regulating DICER1. Song *et al.*^22^ showed that miR-107 was capable of advancing proliferation of GC cells by targeting CDK8. However, Feng *et al.*^23^ reported an opposite role of miR-107 that it was down-regulated in GC and acted as a tumor suppressor to oppose proliferation and invasion of GC cells. In our present study, functional analyses revealed that miR-107 stimulated cell growth, migration, and invasion, in agreement with Li *et al.*^14^ and Song *et al.*^22^, indicating that miR-107 could function as onco-miRNA in GC through multiple targets, including Dicer, CDK8, and NF1. The biological processes and molecular mechanisms underlying miR-107 still remain unclear in GC, and further functional studies are warranted to address these unsolved issues.

NF1 is a GTPase which converts active Ras-GTP to its inactive form, thereby negatively regulating Ras signaling^24^. Loss of NF1 expression by mutation^25^, copy number alteration^26^, or miRNA regulation^18^ can result in constitutive activation of Ras, which can mediate signal transduction via multiple pathways, including Ras/Raf/MEK/ERK pathway, leading to various cancer phenotypes like decreased apoptosis and increased proliferation and migration^27^. It is biologically plausible that the observed miR-107 phenotype in GC may be attributable to ERK activation induced by decreased NF1 expression. Recently, Lenarduzzi *et al.*^18^ reported that miR-193b enhanced tumor progression via down regulation of NF1, which in turn leading to activation of ERK, resulting in proliferation, migration, invasion, and tumor formation.

miRNAs exert their function by repressing translation and/or triggering degradation of mRNA targets^28^. It is reported that miRNA is involved in the ARE-mediated mRNA instability^20^. By bioinformatics analysis, we identified an ARE element adjacent to the second predicted miR-107 target site in *NF1* 3′-UTR. Our results showed that *NF1* mRNA stability was enhanced after miR-107 knockdown. We proposed that miR-107 was involved in the ARE-mediated mRNA instability. Further studies on the role of direct interaction between miR-107 and ARE element in the regulation of *NF1* mRNA stability are warranted. There is a predicted HuR binding site adjacent to the second predicted miR-107 target site in *NF1* 3′-UTR (Fig. 3A). Haeussler *et al.*^29^ found that mRNA binding protein HuR could interact with ARE element in the 3′-UTR of *NF1*, thereby negatively regulating the expression of mRNA on the posttranscriptional level. Therefore, the role of HuR in the regulation of *NF1* mRNA stability by miR-107 also merits further investigation.

Some limitations in this study should be addressed. First, the function of miR-107 was evaluated through miR-107 knockdown in a loss-of-function model. Gain-of-function studies via overexpression of miR-107 in GC cell lines are needed to verify our findings. Second, future *in vivo* studies are needed to validate the role of miR-107 in tumor development and progression. It is reported by Li *et al.* that silencing the expression of miR-107 could suppress the migration and invasion of GC cell in nude mice^30^. We propose that the miR-107 may play an important role *in vivo* in GC carcinogenesis through multiple targets including NF1.

In summary, our data demonstrated that miR-107 targeted NF1, and suppression of miR-107 enhanced proliferation, migration and invasion of GC, whereas repression of NF1 promoted these phenotypes. We propose that miR-107 may serve as a useful therapeutic strategy for advanced GC.

## Additional Information

**How to cite this article**: Wang, S. *et al.* miR-107 regulates tumor progression by targeting NF1 in gastric cancer. *Sci. Rep.* **6**, 36531; doi: 10.1038/srep36531 (2016).

## Figures and Tables

**Table 1:** The relationship between miR-107 expression and clinicopathological feature of 55 GC patients.

Clinicopathological variables	Number of each group	MiR-107 expression		*P* value
		high	low	
Age(years)				
<60	18	8	10	0.465
≥60	37	15	22	
Sex				
Male	42	20	22	0.695
Female	13	7	6	
Tumor size				
≤5 cm	32	12	20	**0.015**
>5 cm	23	15	8	
Tumor site				
Cardia	25	12	13	0.694
Non-cardia	30	16	14	
Histological type				
Diffuse	33	15	18	0.509
Intestinal	22	12	10	
Depth of invasion				
T1+T2	15	4	11	**0.018**
T3+T4	40	25	15	
Lymph nodedistant metastasis				
N0+N1	18	11	7	0.783
N2+N3	37	24	13	
Distant metastasis				
M0	43	22	21	0.659
M1	12	7	5	
TNM				
I+II	16	10	6	0.379
III+IV	39	29	10	

**Table 2:** Computational prediction of miR-107 targets.

Genes	TargetScan5.1	Microcosm Target V5	PicTar^a^	miRanda^b^
NF1	3	54	442	735
UPF2	24	460	655	358
CACNA2D1	72	506	742	289
ZBTB10	141	64	365	98
BAZ2A	148	21	432	21
HTR4	149	373	111	130
KIAA1033	158	559	508	24
KIF23	173	995	732	959
TLK1	186	884	193	38
TGFBR3	219	18	603	165
LRP1B	250	110	589	553
C20orf39	253	137	600	984
SH3GL2	254	8	475	134
RNF125	261	71	748	271
WNT3A	269	337	319	935
DLL1	285	4	445	394
RGS4	290	414	252	216
SYT6	291	474	524	693
OGT	294	1026	2	915
MTMR4	302	920	200	227
VAMP8	357	49	516	839
CCNE1	420	2	324	1441

The figure in the table indicated the rank order of each gene in the respectivemiRNA targets prediction software.

^a^Last updated March 2007.

^b^Last updated June 2005.
